# 

**Published:** 1891-12

**Authors:** 


### Bristol Medico-Chirurgical Journal.

### DECEMBER, 1891.

### CONTENTS.

ORIGINAL PAPERS: *Page*

Medical Progress, Local and General. By F. R. CROSS, M.B. Lond., F.R.C.S. Eng. ... ... ... ... ... ... ... ... ... ... ... ... 241

Epilepsy Treated by Sulphonal. By GILBERT A. BANNATYNE, M.D., C.M. Glasg. ... ... ... ... ... ... ... ... ... ... ... ... 261

HEALTH-RESORTS IN THE WEST OF ENGLAND AND SOUTH WALES ... ... 267

PROGRESS OF THE MEDICAL SCIENCES ... ... ... ... ... ... ... ... ... 278

REVIEWS OF BOOKS ... ... ... ... ... ... ... ... ... ... ... ... ... ... 298

NOTES ON PREPARATIONS FOR THE SICK ... ... ... ... ... ... ... ... ... 309

MEETINGS ... ... ... ... ... ... ... ... ... ... ... ... ... ... ... ... 311

THE LIBRARY OF THE BRISTOL MEDICO-CHIRURGICAL SOCIETY ... ... ... 313

SCRAPS ... ... ... ... ... ... ... ... ... ... ... ... ... ... ... ... 319

